# Challenges in Detecting HIV Persistence during Potentially Curative Interventions: A Study of the Berlin Patient

**DOI:** 10.1371/journal.ppat.1003347

**Published:** 2013-05-09

**Authors:** Steven A. Yukl, Eli Boritz, Michael Busch, Christopher Bentsen, Tae-Wook Chun, Daniel Douek, Evelyn Eisele, Ashley Haase, Ya-Chi Ho, Gero Hütter, J. Shawn Justement, Sheila Keating, Tzong-Hae Lee, Peilin Li, Danielle Murray, Sarah Palmer, Christopher Pilcher, Satish Pillai, Richard W. Price, Meghan Rothenberger, Timothy Schacker, Janet Siliciano, Robert Siliciano, Elizabeth Sinclair, Matt Strain, Joseph Wong, Douglas Richman, Steven G. Deeks

**Affiliations:** 1 San Francisco VA Medical Center (SFVA) and University of California, San Francisco (UCSF), San Francisco, California, United States of America; 2 Vaccine Research Center, National Institutes of Health, Bethesda, Maryland, United States of America; 3 Blood Systems Research Institute (BSRI), San Francisco, California, United States of America; 4 Bio-Rad Laboratories, Redmond, Washington, United States of America; 5 National Institute of Allergy and Infectious Diseases, National Institutes of Health, Bethesda, Maryland, United States of America; 6 Department of Medicine, Johns Hopkins University School of Medicine, Baltimore, Maryland, United States of America; 7 University of Minnesota, Minneapolis, Minnesota, United States of America; 8 Institute of Transfusion Medicine and Immunology, Heidelberg University, Mannheim, Germany; 9 Department of Diagnostics and Vaccinology, Swedish Institute for Infectious Disease Control and Department of Microbiology, Tumor and Cell Biology, Karolinska Institutet, Solna, Sweden; 10 Department of Medicine, University of California, San Francisco (UCSF), San Francisco, California, United States of America; 11 Department of Neurology, University of California, San Francisco (UCSF), San Francisco, California, United States of America; 12 Howard Hughes Medical Institute, Baltimore, Maryland, United States of America; 13 University of California San Diego (UCSD), La Jolla, California, and Veterans Affairs San Diego Healthcare System, San Diego, California, United States of America; Duke University Medical Center, United States of America

## Abstract

There is intense interest in developing curative interventions for HIV. How such a cure will be quantified and defined is not known. We applied a series of measurements of HIV persistence to the study of an HIV-infected adult who has exhibited evidence of cure after allogeneic hematopoietic stem cell transplant from a homozygous CCR5Δ32 donor. Samples from blood, spinal fluid, lymph node, and gut were analyzed in multiple laboratories using different approaches. No HIV DNA or RNA was detected in peripheral blood mononuclear cells (PBMC), spinal fluid, lymph node, or terminal ileum, and no replication-competent virus could be cultured from PBMCs. However, HIV RNA was detected in plasma (2 laboratories) and HIV DNA was detected in the rectum (1 laboratory) at levels considerably lower than those expected in ART-suppressed patients. It was not possible to obtain sequence data from plasma or gut, while an X4 sequence from PBMC did not match the pre-transplant sequence. HIV antibody levels were readily detectable but declined over time; T cell responses were largely absent. The occasional, low-level PCR signals raise the possibility that some HIV nucleic acid might persist, although they could also be false positives. Since HIV levels in well-treated individuals are near the limits of detection of current assays, more sensitive assays need to be developed and validated. The absence of recrudescent HIV replication and waning HIV-specific immune responses five years after withdrawal of treatment provide proof of a clinical cure.

## Author Summary

There is intense interest in developing a cure for HIV. How such a cure will be quantified and defined is not known. We applied a series of measurements of HIV persistence to the study of an HIV+ adult who has exhibited evidence of cure after a stem cell transplant. Samples from blood, spinal fluid, lymph node, and gut were analyzed in multiple laboratories using different approaches. No HIV was detected in blood cells, spinal fluid, lymph node, or small intestine, and no infectious virus was recovered from blood. However, HIV was detected in plasma (2 laboratories) and HIV DNA was detected in the rectum (1 laboratory) at levels considerably lower than those expected in antiretroviral treated patients. The occasional, low-level HIV signals might be due to persistent HIV or might reflect false positives. The sensitivity of the current generation of assays to detect HIV RNA, HIV DNA, and infectious virus are close to the limits of detection. Improvements in these tests will be needed for future curative studies. The lack of rebounding virus after five years without therapy, the failure to isolate infectious virus, and the waning HIV-specific immune responses all indicate that the Berlin Patient has been effectively cured.

## Introduction

Given the well-recognized limitations of antiretroviral therapy (ART)—which include side effects, costs, and difficulties delivering complex regimens to a global population for decades—there is intense interest in curative interventions [1], [2]. This interest in curative strategies is also driven by a single case report in which a cure was apparently achieved [3]. In 2007, an HIV-infected adult living in Berlin developed acute myelogenous leukemia (AML), for which he was treated with an allogeneic hematopoietic stem cell transplant from a donor who was homozygous for the CCR5Δ32 deletion [3], which confers resistance to infection with CCR5-utilizing virus. The patient interrupted ART soon after the transplant and has had no detectable plasma HIV RNA for over five years [3], [4]. Previous studies reported that: 1) he lacked HIV RNA in cerebrospinal fluid (CSF) [4]; 2) he had no detectable HIV DNA in PBMC, bone marrow, brain, or colon [3], [4]; 3) HIV-specific T cell responses decreased after the transplant [3]; and 4) he lost antibodies to Pol and Gag but not Env [3]. Although CCR5-expressing cells were detected in the colon at 5.5 months post-transplant [3], no CCR5-expressing cells were detected in the colon at later time points or in the liver or the brain [4].

Despite the unquestioned success of the transplant, theoretical reasons suggest that HIV could survive the transplant. These include: 1) the possible presence of X4-tropic virus prior to transplant [3], [5]; 2) the detection of rare CCR5+ macrophages 5.5 months after transplant [3]; and 3) the possibility of long lived nonhematopoietic cell reservoirs [6] that could produce virus even if the ability to replicate were constrained by lack of CCR5-expressing hematopoietic cells.

In most ART-suppressed patients, the level of persistent HIV is very low. Even with single copy assays, some patients have essentially no detectable HIV RNA in plasma (i.e., <0.1–1 copy/mL, depending on volume) [7], and only one in approximately a million circulating CD4+ T cells contains replication-competent virus [8], [9], [10], [11], [12]. The burden of HIV may be higher in the lymphoid tissues [8], [13], [14], [15] and gut [16], [17], [18]. As most long-term treated adults have HIV burdens that are near or at the sensitivity of current assays, it is unclear as to whether such assays will be amenable to monitoring virologic responses to future curative interventions.

In 2011, the Berlin Patient transferred his care to San Francisco and consented to a series of studies aimed at determining if HIV persisted. Multiple samples were obtained from a number of different sites, including lymphoid tissues, and relatively large biological inputs were analyzed using the most sensitive assays available. Our objectives were to determine whether the transplant resulted in a sterilizing cure and to assess the capacity of currently available assays to detect and quantify possible low-level persistence.

## Methods

**Figure 1:**
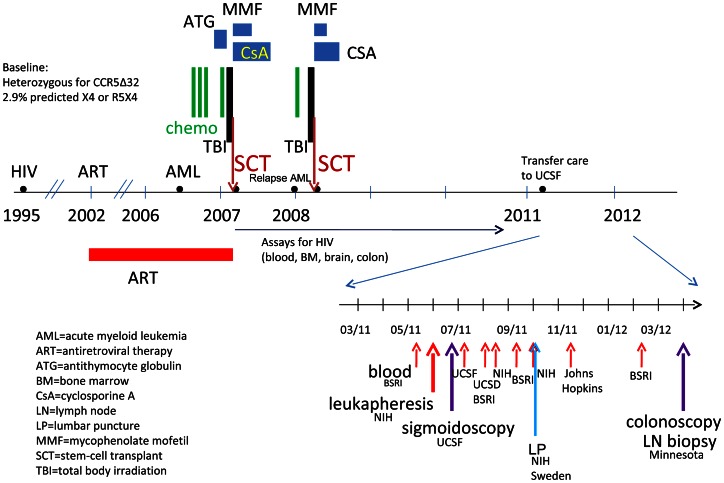
Timeline for clinical treatments and study samples.

The subject was enrolled in the UCSF-based SCOPE cohort and had multiple study visits over two years (Figure 1). Plasma, serum, and PBMC were obtained at each visit. The subject also consented to separate procedures at UCSF, including leukapheresis, lumbar puncture, and flexible sigmoidoscopy with rectal biopsies. He was also seen at the University of Minnesota, where he underwent a lymph node biopsy and a colonoscopy with ileal and rectal biopsies. In order to compare HIV antibody levels in the subject with those from HIV-infected and HIV-uninfected subjects, blood samples were analyzed from participants in another UCSF based pathogenesis cohort, the Options study. All procedures were approved by the Institutional Review Boards at UCSF and the University of Minnesota.

### Samples and labs

Samples (plasma, PBMC, CSF, lymph node, gut biopsies) were analyzed in a number of different laboratories with expertise in detecting extremely low levels of virus, including the Blood Systems Research Institute (BSRI); Johns Hopkins; the National Institutes of Health (NIH); the Karolinska Institutet in Solna, Sweden; the University of California San Diego (UCSD); the University of California, San Francisco (UCSF); and the University of Minnesota.

### Plasma and PBMC

Plasma HIV RNA was measured in 4 different laboratories using 4 different techniques: 1) Roche Ampliprep assay [19]; 2) single copy assay [7], [20] (SCA); 3) Transcription-Mediated Amplification (TMA) [21], [22]; and 4) a modified Abbott assay that involves pelleting virus from 30 ml of plasma [23] (Table 1). Cell-associated HIV DNA in PBMC was measured in 4 laboratories using qPCR [19], [22], [24], [25] or digital droplet PCR [26] (Table 2). Cell-associated HIV RNA in PBMC was measured in 3 laboratories using the Roche Ampliprep [19], qRT-PCR [24] or TMA [22]. Latently-infected peripheral CD4+ T cells (in infectious units per million cells, IUPM) were measured in 2 laboratories by co-culture [27], [28].

**Table 1:** HIV RNA in plasma.

Sample	Date	Lab	Input	Assay	Detection Limit	False Positive Rate^1^	Result
Plasma (3 time points)	5/31/11	NIH	10 ml	Roche Ampliprep [19]	1–2 copy/ml	1/179 (0.6%)	Detected in 3 of 10 replicates (at 1–2 copies)
	8/16/11		24 ml				Detected in 1 of 24 replicates (at 70 copies)
	10/3/11		24 ml				ND^2^
Plasma (1 time point)	7/5/11	SFVA/UCSF	30 ml	Modification of Abbot RealTime (pellet from 30 ml) [23]	<1 copy/ml [23]		ND (<1 copy/ml)
Plasma (1 time point)	11/17/11	Johns Hopkins	17 ml	SCA [7]	0.3 copy/ml [7]	22.2%	Detected in 2 of 6 replicates, each at 4–5 copies
Plasma (4 time points)	5/10/11	BSRI	2.5 ml	TMA [22]	1 copy/ml [22]	6/3515 (0.2%)	ND (<1 copy/ml)
	8/1/11		2.5 ml				ND
	9/7/11		2.5 ml				ND
	2/10/12		2.5 ml				ND

1 Historical, as determined from the number of positive wells out of all wells containing samples from HIV- subjects/donors that had been processed and run along with positive samples.

2 ND = not detected.

**Table 2:** HIV in blood cells.

Sample	Date	Lab	Measure	Input	Assay	Detection Limit	False Positive Rate^1^	Result
PBMC	5/31/11	NIH	HIV DNA	30×10^6^ cells	qPCR for LTR [19]	0.5–2.6 copy/µg	0/72 (<1.4%)	ND^2^
PBMC	7/5/11	SFVA/UCSF	HIV DNA	10^6^ cells	qPCR for LTR [24], [56]	1–2 copy/reaction [56]	1/130 (0.8%)	1 of 10 wells equivocal
PBMC	11/17/11	Johns Hopkins	HIV DNA	10^6^ cells	qPCR for Gag [25]	1 copy/reaction		ND (**≤**1 in 10^6)^
PBMC	8/1/11	UCSD	HIV DNA	10^6^ cells	Digital droplet PCR for Pol [26]	0.52 copy/10^6^ cells	7/29 (24%)	ND (**≤**1 in 10^6^)
PBMC (sorted)	2/29/12	Vaccine Research Center	HIV DNA	6.7×10^4^ CCR5^+^ cells	Fluorescence- assisted clonal amplification of Env	1 copy/reaction		ND
				7.6×10^6^ CCR5^−^ non-T cells				ND
				3.5×10^6^ CCR5^−^ CD4^+^ T cells				ND
				2.1×10^6^ other CCR5^−^ T cells				ND
PBMC	5/31/11	NIH	HIV RNA	10×10^6^ cells	Roche Ampliprep [19]	1–2 copy/ml	1/179 (0.6%)	ND (**≤**1 in 10^7^)
PBMC	7/5/11	SFVA/UCSF	HIV RNA	10^6^ cells	qRT-PCR for LTR [24], [56]	1–2 copy/reaction [56]	0/83 (<1.2%)	ND (≤1 in 10^6^)
PBMC	6/20/11	BSRI	HIV RNA	3×10^6^ cells	TMA [22]	1 copy/ml [22]	6/3515 (0.2%)	ND (**≤**1 in 3×10^6^)
PBMC (sorted)	2/29/12	Vaccine Research Center	HIV RNA	6.8×10^4^ CCR5^+^ cells	RNA sequencing	1 read/sample		ND
				8.6×10^6^ CCR5^−^ non-T cells				ND
				3.4×10^6^ CCR5^−^ CD4^+^ T cells				ND
				4.2×10^6^ other CCR5^−^ T cells				ND
Peripheral CD4+T cells	5/31/11	NIH	Replication compent HIV (IUPM)	1.4×10^9^ cells	Co-culture [27]			ND (**≤**1 IU/10^9^ cells)
Resting peripheral CD4+ T cells	11/17/11	Johns Hopkins	Replication compent HIV (IUPM)	33×10^6^ cells	Co-culture [28]			ND (**≤**1 IU/10^7^ cells)

1 Historical, as determined from the number of positive wells out of all wells containing samples from HIV- subjects/donors that had been processed and run along with positive samples.

2 ND = not detected.

### CSF

Lumbar puncture was performed once (Table 3). The CSF was spun down to yield 8,400 cells, which were tested for HIV DNA in one laboratory [19]. The supernatant CSF was tested for HIV RNA in two laboratories [7], [19].

**Table 3:** HIV in tissues.

Sample	Date	Lab	Measure	Input	Assay	Detection Limit	False Positive Rate^1^	Result
CSF cells	10/3/11	NIH	HIV DNA	8,400 cells	qPCR [19] for LTR	0.5–2.6 copy/µg	0/72 (<1.4%)	ND^2^
CSF	10/3/11	Sweden	HIV RNA	7 ml	SCA [7]	0.3 copy/ml [7]		ND (≤0.1 copy/ml)
CSF	10/3/11	NIH	HIV RNA	10 ml	Roche Ampliprep [19]	1–2 copy/ml	1/179 (0.6%)	ND (≤0.1 copy/ml)
Lymph Node	3/27/12	Vaccine Research Center	HIV DNA	No cell counts available	Fluorescence- assisted clonal amplification of Env	1 copy/reaction		ND
Lymph Node	3/27/12	Vaccine Research Center	HIV RNA	No cell counts available	RNA sequencing	1 read/sample		ND
Lymph Node	3/27/12	Univ. of Minnesota	HIV RNA		ISH [13]	10^3–4^ cells/g		ND
Rectal biopsy	6/20/11	SFVA/UCSF	HIV DNA	DNA from 2.7×10^6^ cells (from 3×2 biopsies)	qPCR for LTR [24], [56]	1–2 copy/reaction [56]	1/130 (0.8%)	2 of 15 wells positive1 in 10^6^ cells
Rectal cells (collagenase digestion)	6/20/11	SFVA/UCSF	HIV DNA	DNA from 1.5×10^6^ cells (out of 2×2.4×10^6^)	qPCR for LTR [24], [56]	1–2 copy/reaction [56]	1/130 (0.8%)	1 of 10 wells positive1 in 10^6^ cells
4 sorted cell populations from rectum	3/27/12	Vaccine Research Center	HIV DNA	3.1×10^5^ CD45^−^ cells	Fluorescence-enabled clonal amplification of Env	1 copy/reaction		ND
				3.1×10^4^ CD45^+^ non-T cells				ND
				1.3×10^4^ CD4^+^ T cells				ND
				3.7×10^4^ other T cells				ND
4 sorted cell populations from rectum	3/27/12	Vaccine Research Center	HIV RNA	8.5×10^5^ CD45^−^ cells	RNA sequencing	1 read/sample		ND
				3.2×10^4^ CD45^+^ non-T cells				ND
				1.3×10^4^ CD4^+^ T cells				ND
				3.8×10^4^ other T cells				ND
Rectal biopsy	6/20/11	SFVA/UCSF	HIV RNA	RNA from 7.8×10^6^cells (from 3×2 biopsies)	qRT-PCR for LTR [24], [56]	1–2 copy/reaction [56]	0/83 (<1.2%)	ND (≤1 in 7.8×10^6^ cells)
Rectal cells (collagenase digestion)	6/20/11	SFVA/UCSF	HIV RNA	RNA from 2.5×10^6^cells (out of 2×2.4×10^6^)	qRT-PCR for LTR [24], [56]	1–2 copy/reaction [56]	0/83 (<1.2%)	ND (≤1 in 2.5×10^6^ cells)
Rectal Biopsy	3/27/12	Univ. of Minnesota	HIV RNA		ISH [13]	10^3–4^ cells/g		Rare signal, ?artifact
4 sorted cell populations from ileum	3/27/12	Vaccine Research Center	HIV DNA	8.6×10^5^ CD45^−^ cells	Fluorescence- enabled clonal amplification of Env	1 copy/reaction		ND (≤1 in 10^6^ cells)
				4.9×10^4^ CD45^+^ non-T cells				ND
				2.9×10^4^ CD4^+^ T cells				ND
				1.0×10^5^ other T cells				ND
4 sorted cell populations from ileum	3/27/12	Vaccine Research Center	HIV RNA	8.8×10^5^ CD45^−^ cells	RNA sequencing	1 read/sample		ND
				5.0×10^4^ CD45^+^ non-T cells				ND
				3.0×10^4^ CD4^+^ T cells				ND
				1.1×10^5^ other T cells				ND
Ileal Biopsy	3/27/12	Univ. of Minnesota	HIV RNA		ISH [13]	10^3–4^ cells/g		ND

1 Historical, as determined from the number of positive wells out of all wells containing samples from HIV- subjects/donors that had been processed and run along with positive samples.

2 ND = not detected.

### Sigmoidoscopy

Thirty rectal biopsies were obtained by sigmoidoscopy. Six biopsies were snap frozen, while 24 biopsies were digested with collagenase [18] to yield total rectal cells, which were counted, aliquoted, and frozen. Total DNA and RNA were isolated from each aliquot of rectal biopsies and rectal cells using Trireagent (Molecular Research Center). RNA was further treated with RNase-free DNase (QIAgen) and purified using QIAgen RNEasy kits. DNA and RNA from each aliquot of rectal biopsies and rectal cells were tested for HIV in one laboratory using qPCR and qRT-PCR for the LTR [24] and normalized to cell equivalents by DNA or RNA mass, as determined by Nanodrop.

### Colonoscopy and Lymph Node (LN) biopsy

Colonoscopy and inguinal LN biopsy were performed at Minnesota. Tissue sections from LN, ileum, and rectum were assessed for HIV RNA by in situ hybridization (ISH) [13]. Sections were taken at 20μ intervals, and two individuals examined each section. The portion of LN that was not placed in fixative was immediately suspended in liquid nitrogen, while the remaining gut biopsies were digested with collagenase to obtain total gut cells. LN tissue and cryopreserved cells from blood and gut were then sent to the NIH, where subsequent procedures included cell sorting (blood and gut), whole transcriptome sequencing, and fluorescence-assisted clonal amplification of HIV *env*.

### Cell sorting

**Figure 2:**
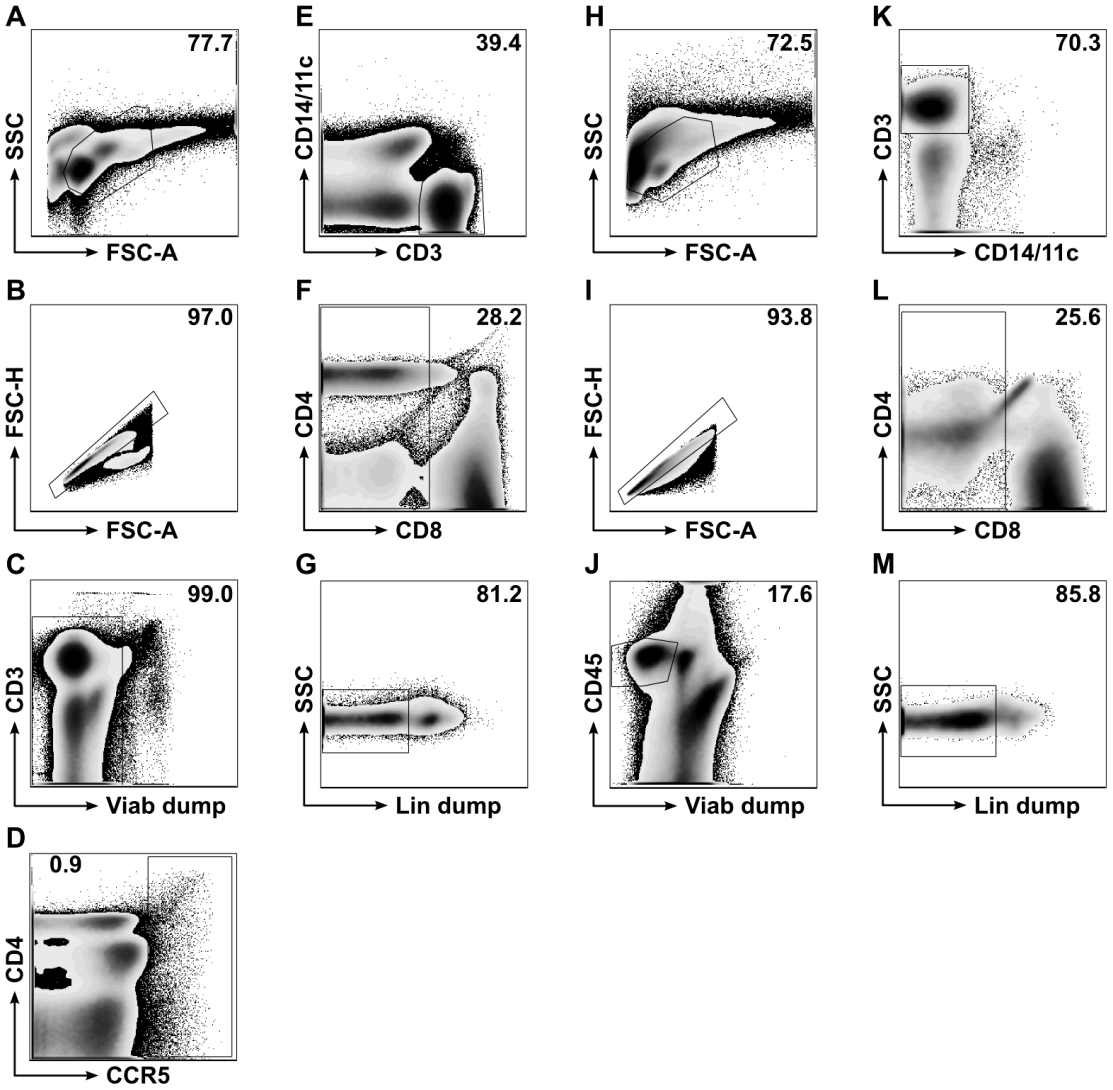
Fluorescence-activated cell sorting (FACS) strategies for PBMC samples (A-G) and GI tract samples (H-M; ileum shown as example). ****For PBMC, all cells were included (panel A), doublets excluded (panel B), and residual non-viable cells excluded by LIVE/DEAD violet cell staining ("Viab dump," panel C). Low-frequency CCR5+ events were then collected in one sorting tube (inside box gate, panel D). Of the remaining, CCR5- events (outside box gate, panel D), CD3+ events negative for CD14 and CD11c were included for further gating (inside polygon gate, panel E), with remaining events collected in a second sorting tube (outside polygon gate, panel E). CCR5-CD3+ events negative for CD14 and CD11c that were also CD8- (panel F) and T cell receptor-γδ-, CD20-, and CD56- ("Lin dump," panel G) were collected in a third sorting tube as presumptive CD4+ T cells. Remaining CD3+ events that were either CD8+ or Lin dump+ were combined in a fourth sorting tube. For ileum and rectum, all cells were included (panel H) and then doublets excluded (panel I). Viable CD45+ events were included for further gating (inside polygon gate, panel J), with all events outside this gate collected in one sorting tube as non-hematopoietic cells. CD3+ events negative for CD14 and CD11c were included for further gating (inside polygon gate, panel K), with remaining events collected in a second sorting tube (outside polygon gate, panel K). CD3+ events negative for CD14 and CD11c that were also CD8- (panel L) and T cell receptor-γδ-, CD20-, and CD56- ("Lin dump," panel M) were collected in a third sorting tube as presumptive CD4+ T cells. Remaining CD3+ events that were either CD8+ or Lin dump+ were combined in a fourth sorting tube. Numbers in upper-right corners of flow plots indicate the percentages of events on plots falling inside gates shown.

PBMC and gut cell suspensions were stained with LIVE/DEAD violet stain (Molecular Probes) and the following fluorescently-conjugated antibodies: CCR5-Cy7PE (Pharmingen); CD45RO-PE-Texas Red (Coulter); CD14-PE (Pharmingen); CD11c-PE (Pharmingen); CD3-H7APC (BD); T cell receptor-γδ-APC (Pharmingen); CD20-APC (BD); CD56-APC (Pharmingen); CD45-Qdot 800 (Invitrogen); CD8-Qdot 655 (Invitrogen); and CD4-Qdot 605 (Invitrogen). Cells were sorted on a FACS Aria into four subsets for each tissue, as shown in Figure 2. Viable single cells from PBMC from two time points were sorted into CCR5+ cells, CCR5- non-T cells, CCR5- T cells that were not CD4+, and CCR5- CD4+T cells. All gut cells from each site were sorted into one of 4 different populations: non-hematopoietic cells (CD45−); hematopoietic cells (CD45+) that were not T cells; T cells that were not CD4+; and CD4+T cells.

### Fluorescence-assisted clonal amplification (FCA) of HIV envelope

Snap-frozen lymph node tissue and sorted cells were homogenized in RNAzol RT (Molecular Research Center, Inc.), and DNA and total RNA were extracted separately. DNA samples were diluted to a concentration of ≤5,000 cell equivalents per well in 384-well plates and amplified with HIV *env*-specific primers ESf2(CCGGCTGGTTTTGCGATTCTAAARTG) and ESr1b(AGGAGCTGTTGATCCTTTAGGTATCTTTC) in PCR reactions containing SYBR Green (Invitrogen). CD4+T cell genomic DNA from a viremic HIV+ subject was amplified as a positive control. HIV *env* amplification was detected by SYBR Green fluorescence and specificity was confirmed by melt curve analysis.

### Whole transcriptome sequencing

mRNA was purified from the sorted samples above as well as PBMC from two HIV- donors, CD4+T cells from one viremic HIV+ control, and stably-infected ACH-2 cells. Messenger RNA was fragmented, and barcoded libraries were constructed and clustered on an Illumina Truseq Paired-End Flowcell v3. The flow cell was sequenced on an Illumina HiSeq 2000 in a 150bp single-read indexed run. Reads were aligned to the human genome (hg19, NCBI Build 37) and transcriptome using TopHat. Unaligned reads were then aligned to the patient's pre-transplant HIV sequences using Novoalign. Reads that did not align to these sequences were then aligned against all molecular HIV clones on the Los Alamos National Laboratories HIV database. In addition, all unfiltered reads were independently aligned to the patient's pre-transplant sequences, the HIV HXB.2 reference sequence, and all molecular HIV clones on the Los Alamos HIV database. Lastly, unfiltered reads were aligned to a wild-type (NM_001100168) and Δ32 CCR5 sequence.

### Immune responses to HIV and other infections

Western blot for HIV was performed on blood from one time point (ARUP labs). Blood from four other time points was analyzed for HIV-specific antibody (Ab) at the BSRI using the HIV-1/2 VITROS assay (Ortho Clinical Diagnostics, Rochester, NY), a detuned version of the HIV-1 VITROS assay [29], and the Limiting Antigen Avidity assay (Sedia, Portland, OR) [30]. To assess how these antibody levels compared with levels in typical HIV infection, we compared the detuned VITROS Ab results with those for participants in the UCSF Options study, whose samples were also tested at BSRI. Results were included from subjects who were HIV- uninfected (negative for HIV antibody and HIV RNA); from subjects who were HIV infected for at least one year and ART-naïve; and from subjects who were HIV infected on suppressive ART for at least 6 months.

Antibodies to other infectious diseases, including CMV, EBV, HSV, VZV, hepatitis B, measles, mumps, rubella, and toxoplasmosis, were measured in post-transplant samples from 5/2011 to 2/2012 using Bio-Rad Bioplex 2200 multiplex and MONOLISA microtiter clinical assays. T-cell responses to HIV and CMV were measured once by flow cytometry for intracellular cytokine staining [31].

## Results

### Plasma HIV RNA

Plasma HIV RNA levels were quantified using several methods. HIV RNA was detected in 2 of 4 laboratories in plasma samples from 3 different time points (Table 1). In one laboratory, HIV RNA was detected by the Roche Ampliprep in 3 of 10 1.0 ml replicates from one time point (each with 1–2 copies, for an average of 0.4 copy/ml), 1 of 24 replicates from a second time point (at 70 copies, for an average of 2.9 copy/ml), and zero of 24 replicates from a third time point. In a separate laboratory, plasma from a fourth time point (17 ml) was positive in 2 of 6 replicates, each with 4–5 copies, for an average of <1 copy/ml. At this level of amplification, false positive signals at the level of 1–2 copies/ml can occasionally be seen, although these controls were negative when this assay was run. No HIV RNA was detected in 2 other laboratories using samples from other independent time points.

### HIV DNA and RNA in PBMC

Unfractionated PBMC samples, each from a different time point, were studied in 4 laboratories using HIV sequence-specific PCR or TMA. No HIV DNA or RNA was detected (Table 2). In a fifth laboratory, PBMC samples from two different time points were sorted into CCR5− CD3− (non-T) cells, CCR5− CD4+T cells, all other CCR5− T cells and, using an inclusive gating strategy to collect all possible events, a cell population that appeared to be CCR5+ by staining (Figure 2a–g). Messenger RNA from each of these sorted populations was negative for HIV by whole transcriptome sequencing, which has been found to yield several hundred HIV-derived reads per sample in comparable experiments on sorted CD4+ T cells from ART-naïve subjects (E. Boritz and D. Douek, unpublished observations). DNA from each of these populations was negative for HIV by fluorescence-assisted clonal amplification of *env*, which detects individual HIV DNA genomes with a sensitivity equivalent to the widely-used quantitative PCR assay for HIV *gag* (E. Boritz and D. Douek, unpublished observations).

### Latent HIV in peripheral CD4+T Cells

Co-cultures were performed in two laboratories using peripheral CD4+T cells from leukapheresis and from venous phlebotomy at a second time point. No replication-competent virus was detected (Table 2).

### HIV in CSF

Lumbar puncture was performed once. No HIV was detected in the CSF supernatant (2 labs) or cells (1 lab) (Table 3).

### HIV in lymph node

One inguinal lymph node was studied in two laboratories (Table 3). No HIV RNA was detected by in situ hybridization or by whole transcriptome sequencing. DNA was negative for HIV by fluorescence-assisted clonal amplification of *env*.

### HIV DNA and RNA in the gut

Whole rectal biopsies and total rectal cells from sigmoidoscopic biopsies were tested for HIV DNA and RNA (Table 3). HIV DNA was detected in DNA isolated from both intact biopsies (2 of 15 wells positive, at 1 and 3 copies) and total rectal cells (1 of 10 wells positive at 1 copy) but not negative controls, while HIV RNA was not detected in either the rectal cells or rectal biopsies.

Biopsies from the ileum and rectum were obtained by colonoscopy at a second time point and analyzed in 2 other laboratories. No HIV RNA was detected in ileal or rectal tissue by ISH or by whole transcriptome sequencing of mRNA from sorted populations of CD45− (non-hematopoietic) cells, CD45+CD3− (non-T) cells, CD4+T cells, or other T cells from ileum and rectum. DNA from each of these populations was negative for HIV by fluorescence-assisted clonal amplification of *env*.

### Sequence data

No sequence data are available from the samples where HIV was detected in the quantitative assays (plasma and rectum). Cloning and sequencing was not attempted from plasma, as no plasma remained from the three time points that were positive for HIV RNA. Likewise, very little rectal DNA remained after repetitive testing by qPCR, and limited attempts to amplify the *env* proved unsuccessful.

Although no HIV was detected in the PBMC by quantitative assays, two labs attempted to amplify and clone *env* from PBMC. Sequencing from one laboratory revealed a subtype B HIV-1 envelope (predicted to be X4) that does not match any known sequence but is also highly divergent from the pre-transplant consensus R5 sequence (16.5% genetic distance). Sequences obtained from PBMC in a second laboratory were consistent with a lab contaminant.

### CCR5 expression

Whole transcriptome sequencing reads from lymph node and sorted subsets of PBMC, ileal cells, and rectal cells were aligned against the CCR5 reference. All 548 reads aligning to the area of the CCR5Δ32 deletion were consistent with the Δ32 sequence of CCR5. Of particular interest were the CCR5 sequences transcribed by the PBMC populations that appeared to express CCR5 protein by flow cytometry. Though these populations were quite sparse (for example, see Figure 2d), they were sorted separately to ensure directed molecular analysis of any potential CCR5-expressing cells. As a result, whole transcriptome sequencing of these populations would have been expected to show abundant CCR5 mRNA had they truly been CCR5+. Instead, CCR5 mRNA was detected at very low levels in these “CCR5+” populations (1.0 and 2.8 CCR5 reads per million total reads in the two sorted PBMC populations, compared to 53.5 and 37.4 CCR5 reads per million total reads in CD4+ T cell populations from ileum and rectum). This suggests that the cells were either not expressing the gene or that the gene transcripts were unstable. Furthermore, all CCR5 reads from the “CCR5+” populations contained the CCR5Δ32 deletion, suggesting that the CCR5 labeling of these cells reflected a fluorescence staining artifact rather true protein expression.

### HIV-specific antibody (Ab)

**Figure 3:**
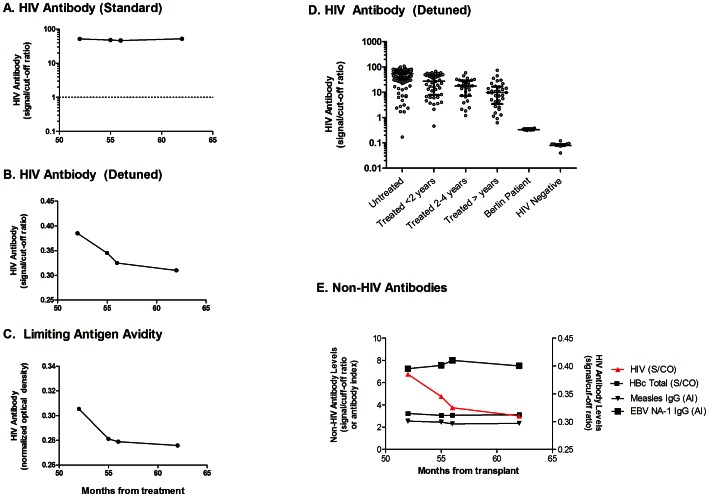
HIV-specific antibodies. Blood from four time points was tested for HIV specific antibody levels using the HIV-1/2 VITROS assay (3A), a detuned version of the HIV-1 VITROS assay (3B), and the Limiting Antigen avidity assay (3C). The y-axis shows the relative level of total HIV-specific antibody, as expressed as the signal to cutoff ratio (3A–B) or normalized optical density, ODn (3C). The x axis represents months since transplant. In Figure 3A, the dotted line represents the diagnostic HIV antibody assay cut-off level used to classify individuals as HIV-positive or HIV-negative. For purposes of comparison, HIV antibody responses were also measured in HIV-uninfected adults, untreated HIV-infected adults, and ART-treated chronically-infected adults using the detuned HIV-1 VITROS assay (Figure 3D). The bars in the scatterplots represent the median and interquartile ranges of distributions of seroreactivity for each group. Finally, samples from the Berlin Patient were tested for antibodies to other infectious diseases (3E). Tests included antibodies to CMV (strong positive, above the limit of detection), EBV, measles, and hepatitis B (all within the range of detection) as well as VZV, mumps, rubella, and toxoplasmosis (all negative, below the limit of detection). Only the results within detectable range of the assay are shown. S/CO = signal/cutoff ratio; ODn = normalized optical density; AI = antibody index.

Western blot was 2+ (strongly positive) for gp160, +/− for p24, and negative for other bands (inconclusive, possibly infected). Blood from four other time points was analyzed using 3 different serological assays. Using the undiluted HIV-1/2 VITROS assay, HIV Ab was readily detectable and relatively stable at all 4 time points, with levels considerably above the cutoff for HIV negative individuals (Figure 3A) but beyond the dynamic range of the assay. In the detuned HIV-1 VITROS assay, HIV specific Ab were detectable at levels that were above those seen in HIV-negative individuals but below those seen in HIV-infected individuals before and after long term suppressive ART (Figure 3D). Moreover, HIV Ab levels tended to decrease over time by both the detuned VITROS and the Limiting Antigen Avidity assays (Figure 3B–C). We screened post-transplant samples for chronic infections using several multiplex commercial assays and identified three viruses (EBV, hepatitis B, and measles) against which the subject had serologic responses that were positive but not above the dynamic range of the assays. In contrast with HIV Ab levels, Ab levels for these viruses remained stable over a 9 month period post-transplant (Figure 3E).

### HIV-specific T cell responses

**Figure 4:**
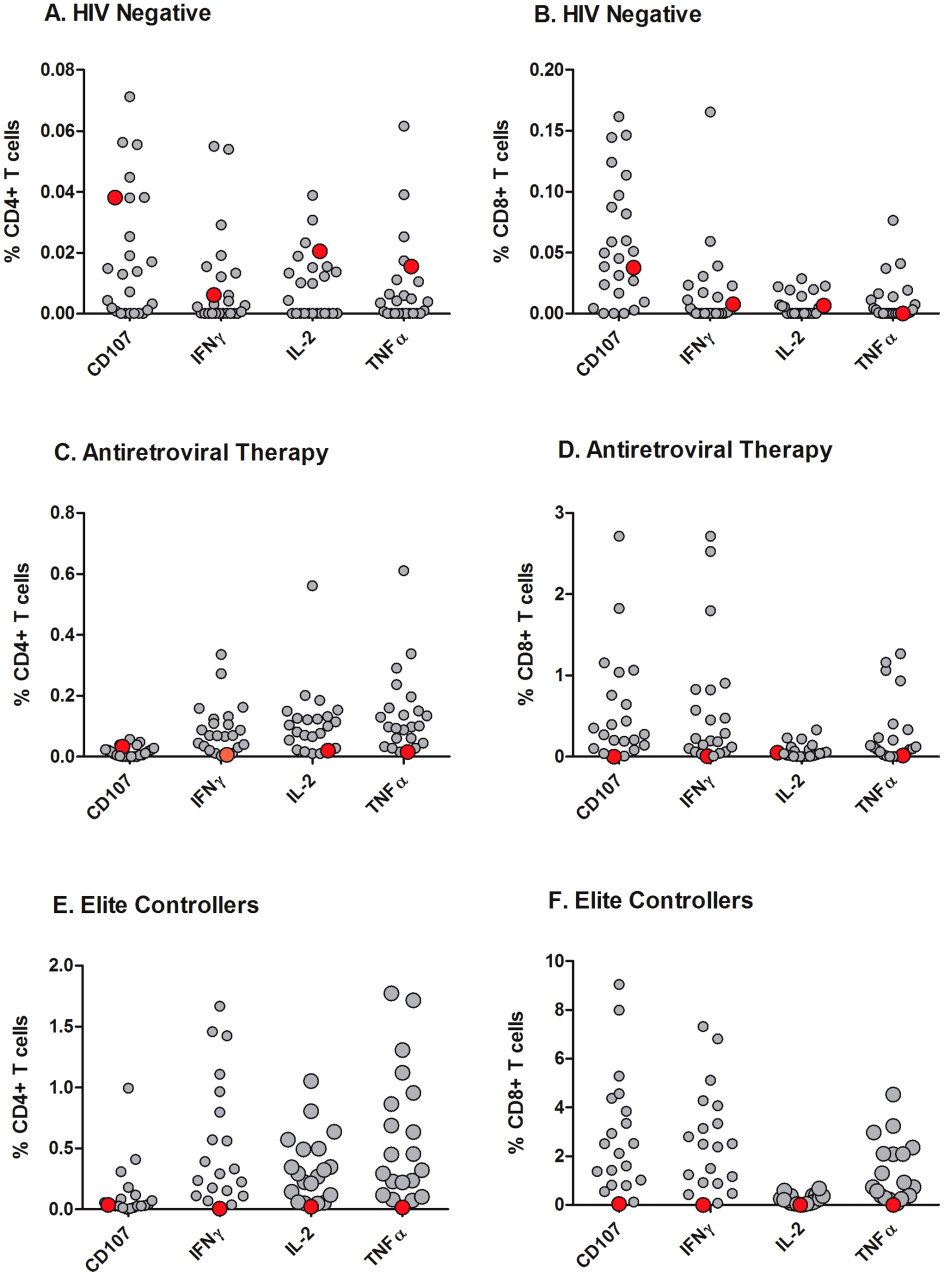
HIV Gag-specific cell mediated immune responses. PBMC were obtained from the Berlin Patient (solid red circles), HIV-uninfected adults (open black circles in 4A–B), chronically HIV-infected adults on long term ART with undetectable plasma viral loads (open black circles, 4C–D), and elite controllers (open black circles, 4E–F). PBMC were stimulated with CMV pp65 or HIV Gag peptide pools, and flow cytometry was used to measure the percentage of CD4+T cells (4A, 4C, 4E) or CD8+T cells (4B, 4D, 4F) with intracellular staining for interferon-γ, tumor necrosis factor-α, IL-2, or CD107. The y axis shows the percent of T cells that express each cytokine in response to HIV Gag. The solid red circle indicates the Berlin Patient, while open black circles indicate individuals from comparator groups.

PBMC were stimulated with CMV pp65 or HIV Gag peptide pools, and flow cytometry was used to measure the frequencies of CD4+ and CD8+T cells with intracellular staining for cytokines. HIV Gag-specific responses from the subject were compared to responses in at-risk HIV-uninfected adults, chronically HIV-infected adults on long term ART with undetectable plasma HIV RNA levels, and chronically infected adults controlling HIV in the absence of therapy (“elite” controllers). The frequencies of T cells expressing or producing CD107, interferon-γ, IL-2 and TNF-α were generally consistent with the levels seen in HIV-uninfected adults (Figures 4A–B), lower than those observed in HIV-infected adults on long-term combination antiretroviral therapy (Figures 4C-D), and much lower than those observed in elite controllers (Figures 4E–F). In contrast, CMV-specific responses were robust and higher than those observed in HIV-uninfected adults (data not shown) [30].

## Discussion

Multiple measurements of HIV persistence were applied to the study of the “Berlin Patient,” who has achieved an apparent cure after a hematopoietic stem cell transplant from a homozygous CCR5Δ32 donor. The vast majority of assays revealed no evidence for persistent HIV, confirming prior reports [3], [4], although 3 independent laboratories detected low level PCR signals in 4 different samples from 2 sites (plasma and rectum). The calculated HIV levels were much lower than typical ART-treated patient patients (Table 4) and close to the detection limit of the most sensitive assays. Replication-competent virus was not detected in PBMC, and HIV-specific antibody (as measured by detuned assays) tended to decline over the 18 months of observation. HIV-specific T cell responses were not detected.

**Table 4:** Summary of virologic measures.

Sample	Measure	# Labs that tested samples	# Labs with + Test	Consensus	Typical levels in ART-suppressed patient	Fold Difference
Plasma	HIV RNA	4	2 labs (3 samples)	?Intermittent positive, ?<1 copy/ml	1–2 copy/ml	2–20
PBMC	HIV DNA	4	0	Negative (≤1 in 10^6–7^)	751 per 10^6^ total PBMC [18]	750–7500
PBMC	HIV RNA	3	0	Negative (≤1 in 10^6–7^)	66 per 10^6^ total PBMC [18]	66–660
Sorted cells from blood	HIV DNA	1	0	Negative	Unknown	
Sorted cells from blood	HIV RNA	1	0	Negative	Unknown	
Peripheral CD4+T	IUPM	2	0	Negative (≤1 IU/10^7–9^ cells)	1 per 10^6^ CD4+T [8], [9], [11], [12]	10–1000
CSF	HIV RNA	2	0	Negative		
CSF cells	HIV DNA	1	0	Negative		
Lymph node	HIV DNA	1	0	Negative	1–12 copies/100 ng [14]	
Lymph Node	HIV RNA	1	0	Negative	≤4 log10 copies/g (FDC) [14]	
Rectum (biopsy or cells)	HIV DNA	2	1	?Intermittent positive, <1 in 10^6^ cells	777 per 10^6^ total gut cells [18]	780
Rectum (biopsy or cells)	HIV RNA	3	0	Negative (<1 in 10^6–7^)	21 per 10^6^ total gut cells [18]	21–210
Ileum (biopsy or cells)	HIV DNA	1	0	Negative (≤1 in 10^6^)	415 per 10^6^ total gut cells [18]	415
Ileum (biopsy or cells)	HIV RNA	2	0	Negative (≤1 in 10^6^)	37 per 10^6^ total gut cells [18]	37

The positive signals can be interpreted in two ways. The first possibility is that all 4 signals could be false positives, perhaps due to nonspecific amplification (e.g., from human endogenous retroviruses) or from contamination of from other clinical samples or lab viruses. The relatively large inputs of biological material and large numbers of replicates may have afforded more opportunities for nonspecific amplification or contamination, especially at copy numbers that are close to the limits of assay detection. Negative controls were run alongside the patient samples for all but the automated Roche assay, and they remained uniformly negative. However, the number of negative controls was generally less than the number of patient wells. For future studies, it may be better to have blinded samples in which the test samples are mixed with an equal or greater number of negative controls.

To better approximate the rate of false positives in a larger number of samples, we used data from other experiments to determine the number of positives among all wells containing samples from HIV-uninfected donors that had been extracted and run along with positive samples. Depending on the number of samples tested, false positives could be documented for most assays (Tables 1–3). In one assay for plasma HIV RNA, the false positive rate of 22% was not much lower than the fraction of positive wells observed for the Berlin Patient (33%), increasing the probability that these may have been false positives. For other assays, the false positive rate was considerably lower than the fraction of positive wells observed for the Berlin Patient, although it is also possible that false positives reactions could occur above the “predetermined” rate.

The second possibility is that one or more of these signals were true positives. If HIV persists at extremely low levels or in nonhomogeneous foci, the detection could be inconsistent and could depend on sampling variability, sample volume, number of replicates, or differences in sensitivities of the various assays. In addition, some assays did not rigorously control for inhibitors of PCR, which can contribute to false negatives. However, even if some HIV DNA or RNA sequence persists, the significance of this finding is uncertain, as most proviral DNA is transcriptionally silent [18], [32] and/or defective [33], [34], and most HIV virions are not infectious [35]. One recent study suggests that the number of resting CD4+ T cells with defective proviruses may be an average of 300 fold higher than the number that can produce replication-competent virus following cellular activation [36].

Despite the PCR signals from plasma, no replication-competent virus could be cultured from circulating blood cells. The vast majority of ART-treated subjects have detectable replication-competent virus using current co-culture assays, particularly if large number of cells are studied, as was the case in this study. Virus isolation is often more challenging in “elite” controllers, however, suggesting these assays have limited sensitivity to detect rare events. Also, it is worth noting that co-cultures would have only assessed for infectious virus in the peripheral CD4+ T cells, which were negative for HIV DNA by PCR, rather than the plasma or gut, where HIV was detected.

The low level of HIV-specific T cell responses, the low and declining levels of HIV-specific Ab in detuned and limiting antigen avidity assays, and the partial seroreversion (based on an indeterminate western blot) are all different from the usual course in antiretroviral-treated adults and “elite” controllers, suggesting very low to absent levels of HIV antigen. Indeed, the levels of residual HIV-specific Ab and T cells detected in this patient were considerably lower than those seen in chronically–infected individuals who have been on suppressive ART for many years, although low or undetectable HIV-specific immune responses have been observed in some patients treated early in the course of HIV infection [37], [38], [39]. Complicating this interpretation is the likelihood that the transplant procedure caused the loss or irreversible impairment of host B and T cells. In HIV-uninfected individuals who have undergone allogeneic stem cell transplant, serum antibodies to tetanus decay at variable rates, but remain detectable in half of patients for a year or more [40]. On the other hand, antibody levels to other infectious diseases, including some associated with latent viral infection, were present at typical levels and stable in the Berlin Patient over a 9 month period post-transplant. Based on these findings we propose that measurement of HIV-specific immune responses may prove to be a sensitive and accurate means to diagnosis an effective cure.

Given the multiple negative signals and the waning HIV-specific immunologic responses, it is possible that all of the positive signals were false positives. True verification of HIV persistence requires sequence confirmation. Unfortunately, the same factors that limit the ability to detect rare, low-level foci of HIV also make it difficult to amplify or clone rare sequences. No sequences are available from the sites where HIV was detected in the quantitative assays (plasma and gut), while the one available sequence comes from the PBMC, where HIV could not be detected in the quantitative assays, and differs from the pre-transplant consensus sequence. Although this sequence does not match any published sequence, it is possible that it is a contaminant from another clinical isolate. Opportunities for contamination may be higher during the nested PCRs and multiple steps used for *env* cloning than they may be in a one step, closed reaction for a quantitative real time PCR.

If one or more PCR signals is a true positive, one must ask why the HIV was not detected previously, and why is it is now detected intermittently. It is possible that HIV persists at extremely low levels, perhaps in rare foci that are frequently missed in sampling. Another possibility is superinfection, especially since the PBMC sequence is predicted to be X4 tropic. Arguing against this possibility is that the vast majority of transmission occurs by one or a limited number of CCR5-utilizing virions [41], [42], HIV infection is extremely rare in homozygous CCR5Δ32 individuals [43], [44], [45], [46], [47], [48], [49], and transmission of X4 virus would be expected to result in sustained replication, which has not occurred.

If HIV persists, one must also question why it has failed to spread, and where it persists. The HIV could be R5-tropic, replication-defective, or unable to overcome his immune defenses. Anatomic sources could include the gut, brain, male genital tract, or lymphoid tissues such as the mesenteric lymph nodes and spleen. Cellular sources could include rare host hematopoietic cells, donor immune cells infected in a non-R5 dependent fashion [50], [51], nonhematopoietic cells (such as neural progenitor cells, astrocytes, neurons, and oligodendrocytes [6]), or cell-associated extracellular virus on the follicular dendritic network [13], [14], [52], [53], [54].

In addition to well-validated PCR, TMA, and *in situ* hybridization assays, we used whole transcriptome sequencing to search for HIV mRNA in the patient samples. Deep sequencing offers several advantages in HIV eradication studies. Because mRNA libraries incorporate all transcripts from each sample, the method's sensitivity is not limited by differences between the sequences of synthetic primers or probes and a patient's autologous viral sequences. In addition, all viral transcripts are quantified simultaneously, allowing characterization of differences in viral gene expression between different cell types, tissues, or individuals. Finally, concurrent sequencing of cellular transcripts allows discernment of host factors, such as CCR5 genotype. It is important to acknowledge that the whole transcriptome sequencing performed here would be expected to detect cell-associated HIV RNA down to a level approximately two orders of magnitude lower than in viremic patients. Therefore, the resulting data do not by themselves rule out a very low frequency of residual HIV RNA-expressing cells in the patient. In future eradication studies, however, greater levels of sensitivity may be achieved by sequencing greater proportions of the mRNA libraries produced. Advances in library preparation and increases in data yield per unit cost may soon make this depth of sequencing routine, allowing detection of single HIV-infected cells within very large cell and tissue samples.

Despite the possibility of intermittently detectable, very low levels of HIV, the Berlin Patient has remained off of ART for 5 years, has no detectable viremia using standard assays, has waning HIV antibody levels, has limited to undetectable HIV-specific T cell responses, and has no evidence of HIV-related immunologic progression. The patient certainly meets any clinical definition for having achieved a long-term remission, and may even have had a sterilizing cure. Even the most extraordinary “elite” controllers described in the literature have more robust evidence for persistent infection [55]. Given that all assays are susceptible to false positive results, and given our inability to provide sequence confirmation, we cannot robustly document HIV persistence and hence conclude he has been cured. This inability to prove a negative is not unexpected, and will likely prove to be problem in future studies on curative interventions. Similar problems are emerging with regard to detection of persistence in other settings, including post-allogeneic stem cell transplants and potentially cured infants. A key priority for the field is the development of robust, well-validated assays that can readily detect very low levels of virus. Future studies of eradication will ultimately require interruption of treatment and subsequent long-term observation as the gold standard for defining a successful outcome.
